# Splenic marginal zone lymphoma in Sweden 2000–2020: Increasing rituximab use and better survival in the elderly

**DOI:** 10.1002/jha2.696

**Published:** 2023-05-03

**Authors:** Henna‐Riikka Junlén, Kristina Sonnevi, Ola Lindén, Mats Hellström, Mariana Villegas Scivetti, Mikael Olsson, Ida Tufvesson, Ann‐Sofie Johansson, Björn Engelbrekt Wahlin

**Affiliations:** ^1^ Division of Hematology Department of Medicine Huddinge Karolinska Institutet Stockholm Sweden; ^2^ Medical Unit Hematology Karolinska University Hospital Stockholm Sweden; ^3^ Department of Oncology Skåne University Hospital Lund Sweden; ^4^ Department of Immunology Genetics and Pathology Uppsala University Uppsala Sweden; ^5^ Section of Hematology and Coagulation Sahlgrenska University Hospital Gothenburg Sweden; ^6^ Section of Hematology Department of Internal Medicine Hallands Sjukhus Varberg Varberg Sweden; ^7^ Division of Hematology Department of Medicine Ryhov County Hospital Jönköping Sweden; ^8^ Cancercentrum Norrlands University Hospital Umeå Sweden

**Keywords:** population‐based, rituximab, splenectomy, splenic marginal zone lymphoma, survival

## Abstract

The treatment of splenic marginal zone lymphoma is debated: splenectomy (the old standard‐of‐care) is better than chemotherapy but maybe not better than rituximab‐containing treatment. We examined all 358 patients diagnosed with splenic marginal zone lymphoma in Sweden 2000–2020. The median overall survival was 11.0 years. The median age was 73 years; 61% were women. Age was the only independently prognostic clinical characteristic. Eighty‐six patients were started on wait‐and‐watch, 90 rituximab monotherapy, 47 rituximab‐chemotherapy, 88 splenectomy, 37 chemotherapy, and 10 both systemic therapy and splenectomy. Overall survival was inferior in patients treated with chemotherapy, but equal in patients treated with rituximab, rituximab‐chemotherapy and splenectomy. Patients treated with both systemic therapy and splenectomy showed good outcome, suggesting that surgery can be safely reserved for nonresponders. After adjustment for age, survival did not differ between patients started on wait‐and‐watch and those treated with splenectomy or rituximab‐containing therapy. Over time, rituximab use and survival increased in patients ≥73 years. This is, to our knowledge, the largest population‐based study of splenic marginal zone lymphoma patients treated with upfront rituximab. We conclude that wait‐and‐watch remains the most reasonable option in asymptomatic splenic marginal zone lymphoma patients. Symptomatic patients should be offered single‐agent rituximab in first line.

## 1 INTRODUCTION

Splenic marginal zone lymphoma is a rare indolent B‐cell neoplasm comprising ∼20% of marginal zone lymphomas and <2% of all lymphomas. The disease involves the spleen, splenic hilar lymph nodes, bone marrow (>95%) and often peripheral blood; lymph nodes outside the abdomen are usually not involved [1]. In the US, the median age at diagnosis is 69 years and the estimated median survival 11.2 years [2]. A diagnosis of splenic marginal zone lymphoma may result from an investigation of splenomegaly or of asymptomatic peripheral lymphocytosis. Symptoms at presentation are mostly related to splenomegaly and cytopaenias. Autoimmune manifestations such as autoimmune haemolytic anaemia or immune‐mediated thrombocytopaenia occur in approximately a fifth of the patients, and an M‐spike, usually IgM, is present in about a third. The diagnostics can be challenging, as an assessment of spleen histology is not always feasible. In these cases, bone marrow histology, blood cytology and immunophenotyping are combined with the clinical picture. Treatment of splenic marginal zone lymphoma is indicated only in symptomatic disease, clinical progression or significant cytopaenias. About a third of the patients never require therapy [3]. There is no consensus on first‐line therapy of splenic marginal zone lymphoma. Modern first‐line treatments include splenectomy [3, 4, 5, 6], rituximab monotherapy [7, 8, 9, 10] and rituximab combined with chemotherapy [9, 10, 11, 12, 13]. Splenectomy is preferable from a diagnostic perspective and gives a rapid relief of symptoms (i.e., abdominal discomfort and cytopaenias related to splenic sequestration) [13]. However, perioperative complications may occur (i.e., infections, bleeding and thrombosis), and patients with splenic marginal zone lymphoma are often elderly and at risk for surgical complications [14]. Cytopaenias secondary to excessive bone marrow infiltration or other extrasplenic manifestations may remain after the surgery. Furthermore, asplenic patients’ lifelong risks of septic infections of encapsulated organisms, particularly pneumococci, are only partially mitigated by appropriate vaccinations [14]. Asplenia is also considered a risk factor in COVID‐19, now an endemic disease, and some countries have prioritized asplenic patients for vaccinations [15, 16]. In the US, rituximab has become increasingly common, especially for frail patients, while splenectomy has become rarer [17]. The current Swedish guidelines for treating splenic marginal zone lymphoma recommend either splenectomy or rituximab as first‐line therapy [18]. We wanted to investigate the outcome in splenic marginal zone lymphoma by first‐line treatment modalities in a large, unselected and relatively recent patient population including many rituximab‐treated patients.

## 2 METHODS

### 2.1 Swedish registries

The Swedish lymphoma register was established in 2000. The Swedish lymphoma register records all new cases of lymphoma in Sweden in patients 18 years or older, with information on subtype, clinical and demographic factors, and first‐line treatment (including the option wait‐and‐watch), as previously described [19]. For the purpose of this study, patient and lymphoma‐specific data were extracted from the Swedish lymphoma register and survival data from the Causes of Death register; a central register for all deaths occurring in Sweden. This study was approved by the Ethics Committee, Stockholm, Sweden (2012/783‐31/3 with amendment 2015/327‐32).

### 2.2 Patients

We identified all patients in the Swedish lymphoma register diagnosed with splenic marginal zone lymphoma between 1st January 2000 and 31st December 2020. Patients who were reported to have, at diagnosis, other extranodal involvement than bone marrow, peripheral blood or liver were excluded, because those features are more congruent with extranodal marginal zone lymphoma (*n* = 11). Patients with nodal involvement outside the abdomen were considered nodal marginal zone lymphoma, and also excluded (*n* = 5). Furthermore, we a priori planned to remove any cases with evidence of primary transformation (within 90 days from the diagnosis of splenic marginal zone lymphoma) but there were none. Finally, patients in whom information on first‐line therapy was missing were excluded. To investigate therapeutic changes over time, the cohort was also analyzed by calendar periods of diagnosis (2000–2005, 2006–2010, 2011–2015 and 2016–2020). Patients were followed from the date of diagnosis to the date of death or last follow‐up (26th August 2022); one patient who had moved abroad was censored at the day she left the country.

### 2.3 Statistical analysis

Depending on the type of variable, we used the Fisher's exact test, the Mann–Whitney–Wilcoxon test, or the Spearman's rank correlation coefficient to investigate relationships between variables. Overall survival was calculated from the date of diagnosis to the date of death from any cause. Patients alive were censored at the day of last follow‐up. Univariate and multivariable Cox proportional hazards analyses were used for survival analysis, always with respect to overall survival. The assumption of proportionality was checked using Schoenfeld's residuals. The *p* values were always two‐tailed, and *p* < 0·05 was considered significant. Stata 14.2 (StataCorp, College Station, TX, USA) was used for all statistical calculations.

## 3 RESULTS

Between 2000 and 2020, a total of 358 Swedish patients were diagnosed with splenic marginal zone lymphoma, without transformation but with information on first‐line treatment decision. The median follow‐up time in survivors was 7.8 years (range, 1.7–22.1 years). At diagnosis, the median age was 73 years (range, 25–95 years; interquartile range, 65–78 years). Age was significantly (*p* = 0·011) higher in the last calendar period 2016–2020 (median 75 years) compared with the earlier periods 2011–2015 (median 72), 2006–2010 (median 72) and 2000–2005 (median 71). Of the 358 patients, 219 (61%) were women, with no sex difference between calendar periods (*p* = 0·75). B symptoms were present in one third, lactate dehydrogenase was elevated in 47%, bone‐marrow involved in 84% and lymphocytosis seen in 50% of the patients (Table 1).

**TABLE 1:** Clinical characteristics at diagnosis and their associations with overall survival.

				Association with overall survival		
Variable		*N*	%	HR	95% CI	*p* Value
Age (years)						<0·00005
	25‐65	97	27	1		
	66‐72	78	22	3·1	1·7–5·5	
	73‐78	99	28	4·9	2·9–8·4	
	79‐95	83	23	9·2	5·4–15·7	
WHO performance status score						0·001
	0	187	54	1		
	1	141	41	1·7	1·2–2·4	
	2‐4	20	6	2·4	1·3–4·4	
Sex						
	Female	219	61	1		
	Male	139	39	0·9	0·7–1·3	0·74
Ann Arbor stage						
	III–IV	310	88	1·2	0·8–2·0	0·41
Lactate dehydrogenase						
	Elevated	140	47	1·0	0·7–1·4	0·90
Bone marrow						
	Involved	302	84	1·1	0·7–1·7	0·59
B symptoms						
	Present	116	33	1·3	1·0–1·8	0·090
Hemoglobin (g/L)						
	<120	138	56	1·5	1·0–2·2	0·083
Lymphocyte count (x 10^9^/L)						
	>4	116	50	1·1	0·7–1·7	0·68
Albumin in serum (g/L)						
	<36	68	29	2·0	1·3–3·1	0·002
Calendar period of diagnosis						0·34
	2000–2005	50	14	1		
	2006–2010	96	27	1·0	0·7–1·6	
	2011–2015	99	28	0·7	0·4–1·2	
	2016–2020	113	32	1·1	0·6–1·8	

Abbreviations: CI, confidence interval; HR, hazard ratio; WHO, World Health Organization.

### 3.1 Treatments

As first‐line strategy, wait‐and‐watch was initiated in 86 patients. Single‐agent rituximab was given to 90 patients. Rituximab‐chemotherapy was used in 47 patients (the chemotherapy combined with rituximab were in 26 patients bendamustine, in 10 CHOP (cyclophosphamide, doxorubicin, vincristine and prednisone) in 4 cyclophosphamide, vincristine and prednisone (CVP), in 5 chlorambucil, in 1 fludarabine‐based and in 1 cladribine). Chemotherapy without rituximab was given to 37 patients (23 chlorambucil, 5 CHOP, 5 cyclophosphamide‐based, 3 fludarabine‐based, 1 bendamustine). Upfront splenectomy was conducted in 88 patients. Another 10 patients underwent a combination of splenectomy and systemic therapy (3 chemotherapy, 4 rituximab monotherapy, 3 rituximab‐chemotherapy); these 10 were analyzed separately (the order of the two interventions could not be discerned in every case). As shown in Table 2, wait‐and‐watch was a rather common approach (24%) throughout the study period, while rituximab monotherapy (25%) and rituximab‐chemotherapy (13%) were very rare initially (2% and 0% of first‐line strategies, respectively) to become dominant by the end (53% and 15%, respectively). Upfront splenectomy (25%) and chemotherapy (10%) showed an inverse pattern, being dominant 2000–2005 (52% and 34%, respectively) but dwindling to become seldom used by 2016–2020 (2% and 1%, respectively). There were also, as expected, age differences in therapy: wait‐and‐watch was more common in the elderly, while upfront splenectomy was more common (36%) in patients ≤65 years and rare (10%) in those ≥79 years (Table 2). Adverse World Health Organization (WHO) performance scores were more common in patients treated with rituximab‐chemotherapy and in those who were started on wait‐and‐watch (on the other hand, the latter showed less B symptoms). There were no differences in age or WHO performance status between the sexes (data not shown).

**TABLE 2:** First‐line treatments and their distributions in clinical characteristics.

		Wait‐and‐watch	Chemotherapy	Rituximab monotherapy	Rituximab‐chemotherapy	Splenectomy alone	Systemic therapy and splenectomy
*N*		86	37	90	47	88	10
Per cent		24%	10%	25%	13%	25%	3%
Calendar period	2000–2005	10%	34%	2%	0%	52%	2%
	2006–2010	26%	10%	6%	11%	43%	3%
	2011–2015	25%	9%	23%	19%	19%	4%
	2016–2020	27%	1%	53%	15%	2%	2%
	*p*	0·079	<0·00005	<0·00005	0·018	<0·00005	0·78
Age category (years)	25‐65	19%	9%	18%	14%	36%	4%
	66‐72	19%	8%	28%	17%	26%	3%
	73‐78	27%	9%	29%	10%	24%	0%
	79‐95	31%	16%	27%	12%	10%	5%
	*p*	0·024	0·18	0·14	0·39	0·0001	0·85
WHO performance status score	0	29%	7%	26%	10%	27%	1%
	1	13%	16%	27%	15%	25%	4%
	2‐4	35%	5%	10%	30%	10%	10%
	*p*	0·016	0·058	0·61	0·031	0·28	0·016
Sex	Women	20%	11%	26%	14%	27%	2%
	Men	30%	9%	25%	12%	21%	4%
	*p*	0·032	0·48	0·90	0·52	0·21	0·20
B symptoms	Absent	31%	9%	23%	11%	22%	3%
	Present	9%	11%	31%	16%	29%	3%
	*p*	<0·0005	0·58	0·12	0·24	0·15	1·00
Bone marrow	Not involved	14%	7%	23%	7%	46%	2%
	Involved	26%	11%	26%	14%	21%	3%
	*p*	0.087	0.48	0.87	0.20	<0.0005	1.00
Hemoglobin (g/L)	≥120	43%	4%	28%	14%	10%	1%
	<120	17%	7%	36%	16%	19%	5%
	*p*	<0.0005	0.40	0.22	0.72	0.073	0.14
Albumin (g/L)	≥36	30%	5%	38%	12%	13%	2%
	<36	22%	9%	26%	22%	13%	7%
	*p*	0.26	0.36	0.13	0.070	1.00	0.050

*Note*: *p*‐Values are for tests of significant differences in the distribution of a treatment type in a clinical characteristic.

Abbreviation: WHO, World Health Organization.

### 3.2 Survival analysis

**FIGURE 1:**
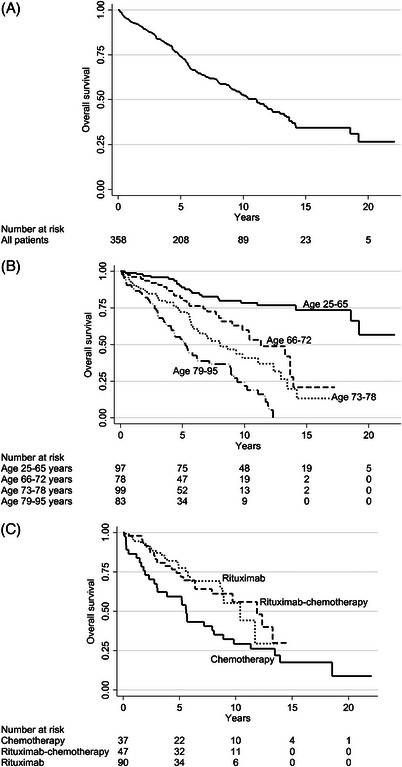
**Kaplan–Meier graphs I**. Overall survival (A) in the entire population of splenic marginal zone lymphoma in Sweden 2000–2020, (B) by age categories, and (C) systemic treatments with and without rituximab.

The estimated median survival was 11.0 years (Figure 1A). High age, poor WHO performance status and low albumin levels were significantly associated with inferior survival in univariate analysis, but calendar period of diagnosis had no impact (Table 1). Patients aged 79–95 years showed a median survival of 5.2 years, those aged 73–78 8.1 years, those aged 66–72 11.3 years, while the median was not reached in patients 25–65 years of age (Figure 1B). All variables showing an association to overall survival with *p* < 0.10 were included in multivariable analysis: age, WHO performance status, albumin levels, and the presence of B symptoms.

**FIGURE 2:**
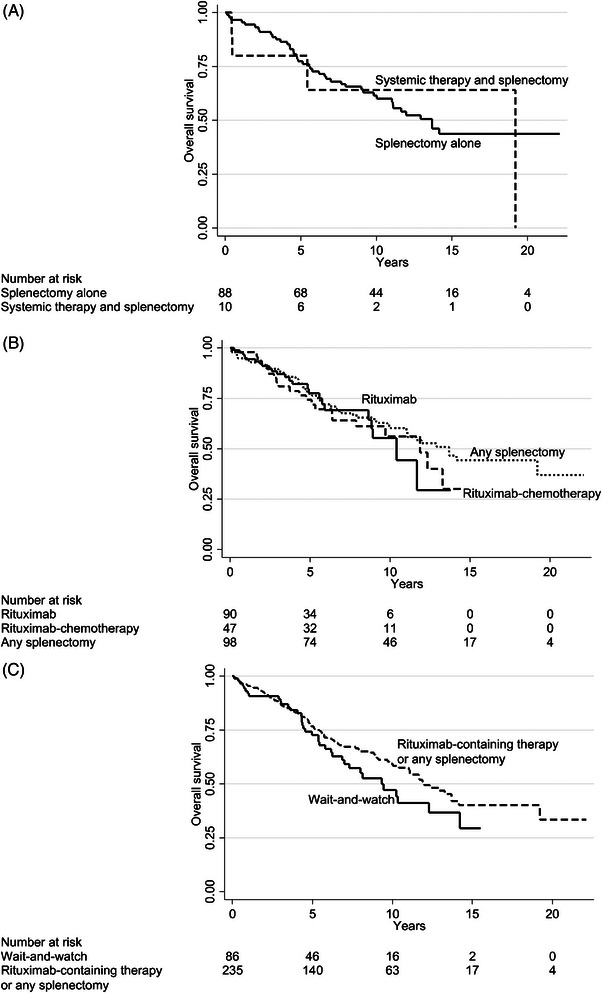
**Kaplan–Meier graphs II**. Overall survival by (A) splenectomy alone versus systemic therapy and splenectomy, (B) splenectomy versus rituximab‐containing treatments and (C) rituximab‐containing therapy or any splenectomy versus wait‐and‐watch.

**FIGURE 3:**
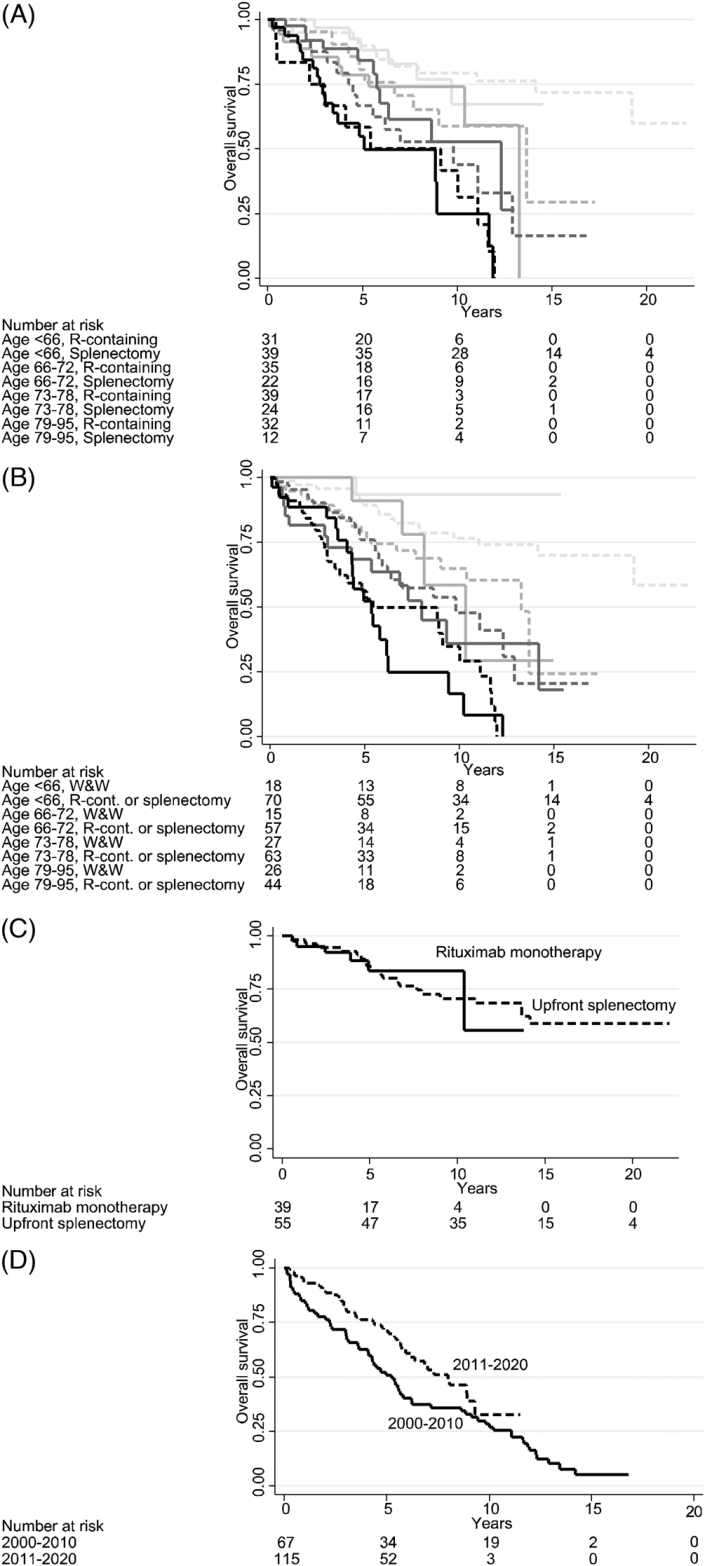
**Kaplan–Meier graphs III: modern therapeutic options and age**. Overall survival graphs. In (A) and (B) ages 25–65 years have light grey, 66–72 medium grey, 73–78 dark grey, 79–95 black lines (solid or dashed). The line patterns represent in (A) solid, rituximab‐containing treatment; dashed, any splenectomy and in (B) solid, wait‐and‐watch; dashed, rituximab‐containing treatment or any splenectomy. (C) Outcome by upfront splenectomy and rituximab monotherapy in patients aged 25–72 years. (D) Outcome by epoch in patients aged 73–95 years.

With respect to overall survival, chemotherapy without rituximab was significantly inferior to rituximab monotherapy and rituximab‐chemotherapy combinations (Figure 1C), also in multivariable analysis (Table 3; multivariable *p* = 0·001). Median survival was 5·6 years in those treated with chemotherapy and 11·7 years in those treated with rituximab‐containing therapy. Chemotherapy was also inferior to splenectomy (Table 3). There appeared to be equally good outcome in the 88 patients who underwent splenectomy alone as in the 10 who received systemic therapy and splenectomy (Figure 2A; Table 3; *p* = 0·70). In a subsequent analysis, all splenectomized patients were grouped as one category (*n* = 98) with an unadjusted median survival of 13·7 years: there were no survival differences between rituximab monotherapy, rituximab‐chemotherapy, and any splenectomy, with adjusted hazard ratios (HRs) between 0·9 and 1·1 and *p* values > 0·6 for all comparisons (Table 3; Figure 2B). The results were identical when the 88 patients treated with splenectomy alone were compared with those given rituximab‐containing treatment (adjusted HR 1·0 and *p* = 0·92; Table 3). Finally, patients who were started on wait‐and‐watch showed a tendency to inferior outcome compared with those who received rituximab‐containing therapy or splenectomy, but only in univariate analysis (Figure 2C); there was no difference in multivariable analysis adjusted for age (HR 1·0; Table 3). We analyzed treatments in different age orders and found that there were no survival differences between rituximab‐containing treatment and splenectomy in any age bracket (Figure 3A), nor were there significant survival differences between wait‐and‐watch and rituximab‐containing treatment/splenectomy (Figure 3B). Rather, Figure 3 shows that with modern therapeutic decisions, encompassing rituximab, and splenectomy, prognosis is mostly driven by age. Figure 3C shows that in patients <73 years, overall survival was similarly excellent after both (*p* = 0.80) rituximab monotherapy and upfront splenectomy (at 10 years 73%). Likewise, there was no overall survival difference between patients ≥73 years treated with rituximab monotherapy and upfront splenectomy (*p* = 0.62), although overall survival as expected was shorter for these (median, 8.9 years). Age remained highly prognostic in all multivariable analyses, but otherwise the only nontreatment factor that retained some independence in multivariable analysis was the presence of B symptoms, which was significant in the models of rituximab‐containing therapy and chemotherapy.

**TABLE 3:** Multivariable models of first‐line therapy with respect to overall survival.

			Univariate analysis		Multivariable analysis		
	*N*	HR	95% CI	*p‐*Value	HR	95% CI	*p‐*Value
A. Chemotherapy versus rituximab‐containing or splenectomy							
Chemotherapy without rituximab	37	1			1		
Rituximab and chemotherapy	47	0·54	0·31‐0·96	0·035	0·50	0·28‐0·90	0·021
Rituximab monotherapy	90	0·50	0·29‐0·88	0·016	0·39	0·22‐0·68	0·001
Chemotherapy without rituximab	37	1			1		
Rituximab‐containing therapy	137	0·54	0·32‐0·83	0·007	0·44	0·27‐0·71	0·001
Chemotherapy without rituximab	37	1			1		
Any splenectomy	98	0·43	0·27‐0·68	0·0003	0·51	0·32‐0·83	0·006
B. Splenectomy alone or combined							
Splenectomy alone	88	1			1		
Systemic therapy and splenectomy	10	1·23	0·44‐3·44	0·70	1·24	0·41‐3·72	0·70
C. Splenectomy versus rituximab‐containing							
Any splenectomy	98	1			1		
Rituximab and chemotherapy	47	1·27	0·75‐2·15	0·37	1·14	0·67‐1·93	0·63
Rituximab monotherapy	90	1·19	0·71‐2·00	0·50	0·90	0·53‐1·52	0·69
Any splenectomy	98	1			1		
Rituximab‐containing therapy	137	1·27	0·82‐1·96	0·27	1·00	0·65‐1·56	0·99
Splenectomy alone	88	1			1		
Rituximab and chemotherapy	47	1·28	0·76‐2·18	0·36	1·14	0·67‐1·96	0·61
Rituximab monotherapy	90	1·22	0·72‐2·06	0·46	0·92	0·54‐1·58	0·77
Splenectomy alone	88	1			1		
Rituximab‐containing therapy	137	1·25	0·81‐1·93	0·32	1·02	0·65‐1·61	0·92
D. Wait‐and‐watch versus treatment							
Any splenectomy or rituximab‐containing therapy	235	1			1		
Wait‐and‐watch	86	1·31	0·89‐1·91	0·17	0·96	0·65‐1·41	0·84

*Note*: All multivariable analyses were adjusted for age, albumin, World Health Organization performance status, and B symptoms.

Abbreviations: CI, confidence interval; HR, hazard ratio.

In the entire population, there were no changes in survival over time, which is explained by the fact that younger patients exerted strong weight in long‐term survival analysis of this indolent disease, and throughout the study period patients <73 years mostly received modern lymphoma treatment (splenectomy or rituximab‐containing regimens): 73% between 2000–2010 and 72% 2011–2020 (Table 4) with an identical survival between epochs (*p* = 0·91). However, in older patients (≥73 years), the fraction of patients receiving modern first‐line treatment increased over time: 46% 2000–2010 and 66% 2011–2020, which was accompanied by significantly better overall survival in the later period among the elderly (*p* = 0·038); their median overall survival improved from 5·2 to 8·0 years (Figure 3D). The increase of modern treatment in the elderly was driven by more use of rituximab‐containing treatment (from 10% to 56%), while splenectomy became rarer (from 36% to 10%), see Table 4.

**TABLE 4:** First‐line treatments divided by median age and epoch.

		Wait‐and‐watch	Chemotherapy	Rituximab monotherapy	Rituximab‐chemotherapy	Any splenectomy	Total
All patients		86 (24%)	37 (10%)	90 (25%)	47 (13%)	98 (27%)	358 (100%)
Patients 25‐72 years		33 (19%)	15 (9%)	38 (22%)	27 (15%)	61 (35%)	175 (100%)
Epoch							
	2000–2010	9 (12%)	12 (15%)	4 (5%)	7 (9%)	46 (59%)	78 (100%)
	2011–2020	24 (25%)	3 (3%)	35 (36%)	20 (21%)	15 (15%)	97 (100%)
Patients 73‐95 years		53 (29%)	22 (12%)	51 (28%)	20 (11%)	36 (20%)	182 (100%)
Epoch							
	2000–2010	21 (31%)	15 (22%)	3 (4%)	4 (6%)	24 (36%)	67 (100%)
	2011–2020	32 (28%)	7 (6%)	48 (42%)	16 (14%)	12 (10%)	115 (100%)

*Note*: One patient in the register was of unknown age at diagnosis. She was upfront splenectomized. This is why the sum of patients splenectomized aged 25–72 and 73–95 years is 97, not 98.

## 4 DISCUSSION

This study presents the largest cohort of splenic marginal zone lymphoma patients treated upfront with rituximab‐containing therapy (*n* = 137), of whom 90 received rituximab as a single agent. The follow‐up times are sufficient for meaningful interpretation (median, 7·8 years). Patients who were treated with chemotherapy alone showed inferior survival compared to those who received rituximab‐containing therapy or splenectomy, agreeing with earlier smaller series [9, 10]. However, we saw no survival differences between patients treated with rituximab‐containing treatment or splenectomy. Some authors have shown better long‐term outcome after rituximab [10, 20], others after splenectomy [13]. All three studies included few patients treated with rituximab or rituximab combinations: 43, 58 and 69 patients, respectively. Interestingly, the last study by Sima et al. used the same register as ours [13], but investigated the period 2007–2017 (during which rituximab was used more selectively) than during the later times covered in our study (when rituximab monotherapy was considered standard of care). It should be noted that first‐line rituximab monotherapy for young patients was not accepted in all of Sweden's healthcare regions until 2017. Herein probably lies part of the explanation why our study shows no survival difference between rituximab‐treated and splenectomized patients. However, also in the report by Sima et al., overall survival times with rituximab monotherapy and splenectomy appear very similar; their main finding was different outcomes between splenectomy versus all other modalities combined (including systemic treatment without rituximab) [13]. As in their material, an overall survival analysis of our material in which splenectomy is compared with all other modalities combined (including systemic treatment without rituximab) would show superiority for splenectomy with *p* = 0·022. As expected, rituximab became more, and splenectomy less, common over the study period, with no overall change in survival. Still, the advent of rituximab allowed for a good treatment option for all patients, including the elderly, in whom rituximab use and overall survival increased.

Wait‐and‐watch remains a good strategy for asymptomatic patients, since there appears to be at least equal survival after adjustment for age, which is the strongest prognostic factor in this disease. The study also shows that in an unselected patient population, the median age is well above 70 years and the female‐to‐male ratio almost 2:1, making this a disease of elderly women. The median survival time in this study is 11·0 years and the median age 73. This suggests that with modern management of this disease, at least in elderly patients, the lymphoma will probably seldom affect their expected survival. Our results agree with previous reports that show equal outcome between splenectomy and rituximab‐containing therapy in patients >65 years [21]. Our study also suggests that wait‐and‐watch remains the most sensible approach to asymptomatic patients. Indeed, in younger patients, survival is excellent with a management using wait‐and‐watch (Figure 3B).

In spite of the inherent bias in a retrospective analysis of different treatments, it appears that rituximab monotherapy, rituximab‐chemotherapy and splenectomy are equal first‐line approaches with respect to overall survival. It is also noteworthy that patients who in first line underwent both splenectomy and systemic treatment (where the first given of these two was presumably suboptimal) had good prognosis, suggesting that one does not lose anything from starting with rituximab monotherapy (preferably preceded by pneumococcus vaccinations). In the small fraction of poor rituximab responders [8], salvage splenectomy appears to be a good second‐line option.

Our study has limitations. We do not know these patients’ hepatitis C status. However, hepatitis C‐induced splenic marginal zone lymphoma would be exceedingly rare in Sweden, because of the low national prevalence of the virus; indeed, in the previous report where the Swedish lymphoma register was used there was not a single case of active hepatitis C in 289 investigated splenic marginal zone lymphoma patients [13]. Another weakness is that there is no information on relapses or second‐line therapy, and thus no information on time to next treatment or progression‐free survival. But those endpoints are less interesting when rituximab monotherapy is compared with rituximab‐chemotherapy or with surgery.

We conclude that wait‐and‐watch is the most reasonable option in unsymptomatic splenic marginal zone lymphoma patients, and those who have symptoms should be offered single‐agent rituximab as a standard first‐line regimen. Although splenectomy shows the same long‐term survival, the complications and lifelong infectious risks after the procedure suggest that it should be reserved for patients who do not respond to rituximab.

## AUTHOR CONTRIBUTIONS

HRJ and BEW planned the study. HRJ and BEW analyzed data and wrote the manuscript. KS, OL, MH, MVS, MO, IT, AJ, and BEW contributed to the registry and data interpretation. All authors participated in the final version of the manuscript.

## CONFLICT OF INTEREST STATEMENT

The authors declare that there is no conflict of interest that could be perceived as prejudicing the impartiality of the research reported.

## FUNDING INFORMATION

The funding source did not affect any part of the research.

## Data Availability

None.
